# Management of Weight Loss in People With Cancer

**Published:** 2016-04-01

**Authors:** Kristy K. Hager

**Affiliations:** Wellmont Cancer Institute in Kingsport, Tennessee

Pathophysiologic changes in people with cancer can lead to anorexia and weight loss. Advanced practitioners who understand this problem can help patients maintain nutritional status and weight, according to Kristy K. Hager, MS, RD, CSO, LDN, an oncology dietitian at Wellmont Cancer Institute in Kingsport, Tennessee.

"Nutrition is extremely important in the cancer treatment and recovery process," said Ms. Hager, who spoke at JADPRO Live at APSHO. "Oncology treatment can overwhelm even a healthy person’s nutritional reserve, and malnutrition can have a profound effect on the cancer patient."

Some 40% to 80% of cancer patients will experience malnutrition at some point during treatment, and a loss of as little as 6% of body weight can predict reductions in treatment response, survival, and quality of life (Bruera, 1997; Shike, 1996).

A balanced diet provides the six basic nutrients important for good health: carbohydrates, protein, fat, vitamins, minerals, and water, as well as adequacy of nutritional intake. These are necessary for body tissue growth and repair, daily energy, fighting off diseases and infections, and treatment and recovery.

## THE IMPORTANCE OF CARBOHYDRATES

A consistent source of carbohydrates in the diet is necessary to eliminate muscle loss and immune compromise.

"Patients tell me they want to stop eating sugar because it feeds their cancer," Ms. Hager said. But glucose, the form of sugar most used in the body, is required by all cells for fuel (not just cancer cells).

"The idea that sugar feeds cancer cells can overwhelm patients with anxiety and lead them to avoid all carbohydrate-containing foods. This is counterproductive, especially when the patient is struggling to maintain weight and manage the side effects of treatment," she pointed out. 

Practitioners can ease fears by counseling patients to "increase color in their diet" and limit processed food. The American Institute for Cancer Research developed "The New American Plate Method," which provides recommendations for healthy eating during and after cancer treatment (www.aicr.org, www.oncologynutrition.org).

## MALNUTRITION SCREENING

Nutrition screening and surveillance can identify patients who are experiencing or at risk for malnutrition. While all cancer patients can benefit from this, persons with gastrointestinal and head and neck cancers are at the highest risk for weight loss and malnutrition.

Malnutrition is defined as undernutrition and changes in body composition occurring due to the cancer itself or from the impact of treatment. It is frequently underreported or ignored, but it can precipitate an array of treatment- and disease-related complications.

Nutrition interventions that prevent or limit malnutrition can positively influence treatment schedules and outcomes. Dose reductions and holding or terminating treatment all have negative impacts; therefore, it is vital that patients maintain performance status and weight, as those factors may determine decisions about treatment.

Early intervention is mandatory, as attempts to reverse severe malnutrition in the oncology patient are challenging and often not successful. Ms. Hager recommends that cancer patients be screened upon admission to the oncology service and routinely throughout the treatment regimen. She recommended choosing a screening tool that is easy to use, rapidly administered, and valid for the intended population.

## CANCER CACHEXIA

Early recognition is important for preventing cancer cachexia, as severe malnutrition progresses to this. "Not all malnourished patients are cachexic, but all cachexic patients are malnourished," said Ms. Hager.

Cancer cachexia is diagnosed in up to 80% of persons with advanced cancer, and is cited as a factor in up to half of all cancer deaths. Once it is established, cachexia is not fully reversible.

"Regardless of the presence of cachexia, the causes of decreased intake need to be addressed, and intervention is required regarding factors and behaviors that can be manipulated," she said.

## CHANGING EATING PATTERNS

A formal nutrition assessment helps to evaluate the characteristics of the malnutrition, and will help in developing a plan for modifying or preventing the underlying factors. What is often revealed is inadequate intake of calories and protein.

While healthy people can easily "clean their plates," cancer patients perceive food differently and may become overwhelmed by the volume of food in front of them. The tendency of family members to "love them through it" by making elaborate meals, filling plates, and even force-feeding is "counterproductive and creates more stress," she said.

Instead, she recommended changing the eating pattern toward small, frequent meals and bites and sips, an approach that has proven successful (Cooper, Burden, Cheng, & Molassiotis, 2015). Small single servings require less energy to consume and also help combat fatigue. Eating in pleasant surroundings can also help with poor appetite. "Remove all caretaking items from the eating area, including bedpans and emesis basins. No one wants to eat around that," she commented.

Early satiety can be managed by separating food and liquid intake. "Unless your patient requires liquids to help them swallow, I recommend limiting fluids with meals to half a cup and drinking other liquids 2 hours before or 2 hours after eating," she stated.

Patients who tolerate liquids better than solids should consume liquids that are calorically dense and nutritionally adequate, she added.

## TIPS FOR SPECIFIC CONDITIONS

Advanced practitioners should anticipate treatment-related side effects that may impact nutritional intake and weight status, and take steps to mitigate them (Table).

**Table:**
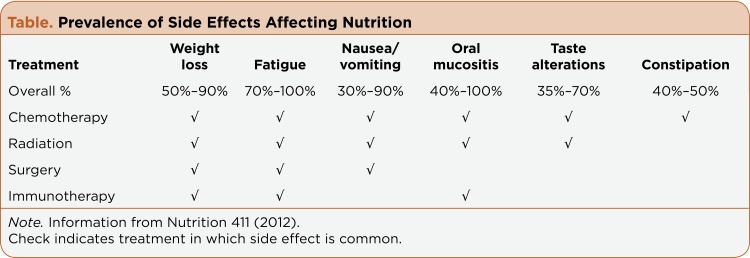
Prevalence of Side Effects Affecting Nutrition

For dysphagia, the most helpful nutrition management strategy is alteration of food and drink. Focus on soft, blended, moist foods from all food groups. It is also imperative to confirm the safety of oral intake, by encouraging double swallows and verbalization after swallowing, Ms. Hager said.

For dry mouth, patients should concentrate on foods with high water content and maintain proper daily oral hygiene. For oral mucositis, soft non-acidic foods that are cold or room temperature may be well tolerated; the use of a straw can aid swallowing and can divert liquids to less painful areas of the mouth.

To deal with taste and smell alterations, rule out mucositis and replace metal silverware with plastic to avoid exacerbating the metallic taste. Sippy cups can divert odors from the patient’s nose. Tart, sour, and acidic foods are often preferred by these patients.

To deal with nausea and vomiting, confirm that patients are taking their antinausea medication as prescribed, then recommend that patients consume small portions, eat slowly, and avoid tight clothing and hot, stuffy atmospheres. When feeling nauseated, they should avoid their favorite foods to prevent the development of new food aversions.

Constipation can often be ameliorated with a high-fiber diet, but limit the increase in fiber to ≤5 g/day and increase fluid concomitantly. For diarrhea, patients must maintain hydration and consume a low-fiber diet (< 13 g/day), with fiber slowly resumed once diarrhea abates.

For hypomagnesemia, dose and route of magnesium repletion should be based on the degree of deficiency and the severity of clinical signs. 

To access evidence-based recommendations and adequate nutritional resources in the absence of a certified specialist in oncology or a registered dietician, advanced practitioners may find the following resources helpful:

- The Oncology Nutrition DPG of the Academy of Nutrition and Dietetics (www.oncologynutrition.org)
- The Academy of Nutrition and Dietetics (www.eatright.org)
- The American Institute for Cancer Research (www.aicr.org)
- The National Cancer Institute (www.cancer.gov)
- www.nutrition411.com
- www.chemocare.com
